# Evaluating the Causal Effects of ADHD and Autism on Cardiovascular Diseases and Vice Versa: A Systematic Review and Meta-Analysis of Mendelian Randomization Studies

**DOI:** 10.3390/cells14151180

**Published:** 2025-07-31

**Authors:** Piotr Ryszkiewicz, Barbara Malinowska, Magdalena Jasińska-Stroschein

**Affiliations:** 1 Department of Experimental Physiology and Pathophysiology, Medical University of Białystok, ul. Mickiewicza 2A, 15-222 Białystok, Poland; barbara.malinowska@umb.edu.pl; 2 Department of Biopharmacy, Medical University of Łódź, ul. Muszyńskiego 1, 90-151 Łódź, Poland

**Keywords:** mendelian randomization, autism spectrum disorder, attention-deficit hyperactivity disorder, cardiovascular diseases, genetic causality, systematic review, meta-analysis

## Abstract

Attention-deficit/hyperactivity disorder (ADHD) and autism spectrum disorder (ASD) are neurodevelopmental disorders with lifelong functional implications. Their potential role as emerging risk factors for cardiovascular diseases (CVDs) is increasingly acknowledged. The aim of this study was to conduct a comprehensive evaluation and meta-analysis of Mendelian Randomization (MR) studies exploring the causal effects of ADHD and ASD on various cardiovascular outcomes and vice versa. Three databases were searched, study quality was evaluated using a STROBE-MR checklist, and relevant data were extracted. In total, 14 studies revealed genetic associations between ADHD or ASD susceptibility and selected CVDs and vice versa. Notably, genetic markers for ADHD were linked to an increased risk of coronary artery disease, heart failure, and various types of stroke. Genetic predisposition to ASD raised the likelihood of atrial fibrillation and heart failure. Atrial fibrillation showed a causal relationship with elevated ADHD risk. Interestingly, hypertension was not associated with ADHD or ASD at the genetic level. Further efforts are needed to fully elucidate the basis of causal links from a mechanistic perspective. Overall, the results highlight the need for cardiovascular risk assessment and management in the clinical care of individuals with ADHD and ASD.

## 1. Introduction

Neurodevelopmental disorders (NDDs) refer to a group of complex conditions that typically emerge early in the developmental period. They are often linked to a range of neuropsychiatric symptoms, with the most common including autism spectrum disorder (ASD), attention-deficit/hyperactivity disorder (ADHD), intellectual disability, communication disorders, specific learning disorders, and motor disorders [1]. They exhibit considerable genetic and clinical diversity [1,2]. Nearly 140 million individuals are affected with ADHD, and over 60 million with ASD globally [3,4]. In recent years, increasing attention has been paid to the co-occurrence of NDDs with various physical health conditions, particularly cardiovascular diseases (CVDs), which may pose a significant health risk for individuals with ADHD and ASD [2,5,6]. Depending on the analyzed population, the prevalence of CVDs among adults with ADHD and ASD is estimated at ~20% [7] and ~40%, respectively [6]. Numerous systematic reviews and meta-analyses of the observational studies have examined the associations between NDDs (particularly ADHD and ASD) and cardiovascular outcomes. Findings from the last five years suggest that both ADHD [5,8,9,10] and ASD [11,12,13] are linked to a higher risk of CVDs, including congenital heart disease (CHD), hypertension, and arrythmias. Conversely, the reverse association suggests that CVDs may also be linked to a higher risk of developing ADHD [5,14,15,16] and ASD [14,16,17,18,19].

While these studies have provided valuable insights into the strength and consistency of these associations, their ability to determine the causality of the observed relationships is limited. The evidence derived from observational studies, despite sophisticated statistical adjustments, remains vulnerable to confounding, reverse causality, and other sources of bias [5]. As a result, the underlying mechanisms linking these conditions remain poorly understood, and it is still unclear whether ADHD or ASD directly contributes to increased cardiovascular risk [2,20]. Possible explanations for why individuals with ADHD may face a heightened risk of developing CVDs refer to a combination of genetic and environmental factors, such as immune system dysregulation, neuromodulatory imbalances, and hypothalamic–pituitary–adrenal (HPA) axis disturbances, as well as behavioral and clinical factors, including unhealthy lifestyle habits, psychiatric comorbidities, and potential cardiovascular effects of stimulant medications [5]. Factors contributing to the high rates of CVD risk among autistic individuals may mirror those identified in other populations, such as elevated perceived stress, inadequate sleep, and the use of antipsychotic medications [6].

Recently, large-scale genome-wide association studies (GWASs) have substantially advanced our understanding of the genetic architecture underlying NDD and CVD risk factors [21,22]. By scanning millions of genetic variants across the genomes of hundreds of thousands of individuals, GWASs have identified numerous single nucleotide polymorphisms (SNPs) associated with complex traits, such as ASD, ADHD [23], hypertension [24], arrhythmias [25], congenital heart disease [26], and others. While these statistical associations offer valuable insights into disease biology, they do not, in themselves, establish causality [27]. Distinguishing causal relationships from non-causal correlations remains a critical challenge in observational epidemiology, particularly given the pervasive influence of confounding and reverse causation [21,28,29].

To address this challenge, the Mendelian Randomization (MR) design has emerged as a powerful analytical framework that leverages genetic variants as instrumental variables (IVs) to infer causal relationships between risk factors and disease outcomes [30,31]. MR capitalizes on the random allocation of alleles during meiosis and fertilization—a process that mimics the randomization in clinical trials—thereby reducing the likelihood of confounding by environmental or behavioral factors [21]. When valid genetic instruments are available, MR can provide evidence about whether an observed association reflects a true causal effect. For an MR analysis to yield valid inferences, three key assumptions must be satisfied: (1) relevance—the genetic variant must be robustly associated with the exposure of interest; (2) independence—the variant must be independent of confounders of the exposure–outcome relationship; and (3) exclusion restriction—the variant must influence the outcome solely through the exposure and not via alternative pathways [31].

This systematic review aims to synthesize the existing evidence from MR studies that explore the causal links between ASD, ADHD, and various cardiovascular outcomes. Specifically, we seek to (1) evaluate the methodological quality and consistency of these studies; (2) summarize the current MR findings on causal relationships between ASD/ADHD and CVDs; and (3) identify gaps in the literature and areas for future research.

## 2. Materials and Methods

### 2.1. Literature Search and Data Extraction

The literature search was performed according to the Preferred Reporting Items for Systematic Review and Meta-Analysis (PRISMA) guidelines [32]. Three databases (PubMed, Scopus, and Web of Science) were searched for relevant citations from inception to 9 April 2025, utilizing the queries “mendelian randomization”, “ADHD” or “attention-deficit hyperactivity disorder”, or “autism”, “autism spectrum disorder”, “ASD”, combined with the names or abbreviations of cardiovascular conditions considered in this review (i.e., hypertension, congenital heart disease, myocardial infarction, stroke and its subtypes, heart failure, coronary heart disease, and arrythmia, including atrial fibrillation)—for details, see Supplementary Table S1. The screening process was performed by two researchers, and any discrepancies about the eligibility of selected articles were resolved through consultation with a third researcher.

Data extraction involved the following: first author’s name, publication year, and population components: study ethnicity, cohort (consortium responsible for genomics research) and sample size, exposures, outcomes, and major findings. The odds ratio (OR) with corresponding 95% confidence intervals (±95% CI) as a result of various MR methods (inverse variance weighted (IVW), weighted median, weighted mode, MR-Egger, MR-PRESSO, and other analyses) was also extracted with an indication of the main (primary) analysis.

### 2.2. Study Quality

The Strengthening the Reporting of Observational Studies in Epidemiology for Mendelian Randomization (STROBE-MR) was used to assess the methodological quality of papers that were included in the meta-analysis [29]. The quality assessment scores were converted into percentages, and then they were categorized into a high risk (scores less than 75%) and medium risk (scores between 75% and 85%) of bias, while scores exceeding 85% were regarded as a low risk of bias. The criteria for assessing the risk of bias of selected papers followed several assumptions: (1) relevance (assumption 1: “the genetic variant must be associated with the exposure”: strength of the instruments); (2) independence (assumption 2: “the genetic variant should not associate with potential confounders in the exposure–outcome association”: whether the genetic association with confounders was evaluated and ethnically homogenous populations were used); (3) exclusion restriction (assumption 3: “the genetic variant should influence the outcome only via the exposure”: whether the study used different MR analytic methods that rely on different assumptions and whether the study evaluated potential pleiotropy). Other considerations were if the study conducted showed a sufficient statistical power (>80%).

Two researchers independently assessed the quality and bias of the selected articles, and any discrepancies were resolved by a consensus with the participation of a third researcher.

### 2.3. Analysis

The forward analysis concerned the association between the genetic risk of neurodevelopmental disorders—attention deficit hyperactivity disorder (ADHD) and autism spectrum disorder (ASD)—as an exposure and the risk of developing cardiovascular diseases (outcome). In the backward analysis, we analyzed the causal relationships between cardiovascular diseases (exposure) and neurodevelopmental disorders (outcome). In both cases, effect size is expressed as odds ratio (OR) ± 95% CI. The heterogeneity between studies was assessed employing I^2^, where a value between 25% and 50% was considered as mild heterogeneity, 50–75% as moderate, and exceeding 75% as severe heterogeneity. Another measure of heterogeneity between studies was Cochrane Q statistics, with *p* > 0.05 suggesting the absence of heterogeneity. A two-sided *p*-value below 0.05 was considered statistically significant. In cases where 2 or more studies used data from the same source or biobank, only the larger one was included in the quantitative meta-analysis. The analyses were performed using STATISTICA Software (13.3).

## 3. Results

### 3.1. Data Search

**Figure 1:**
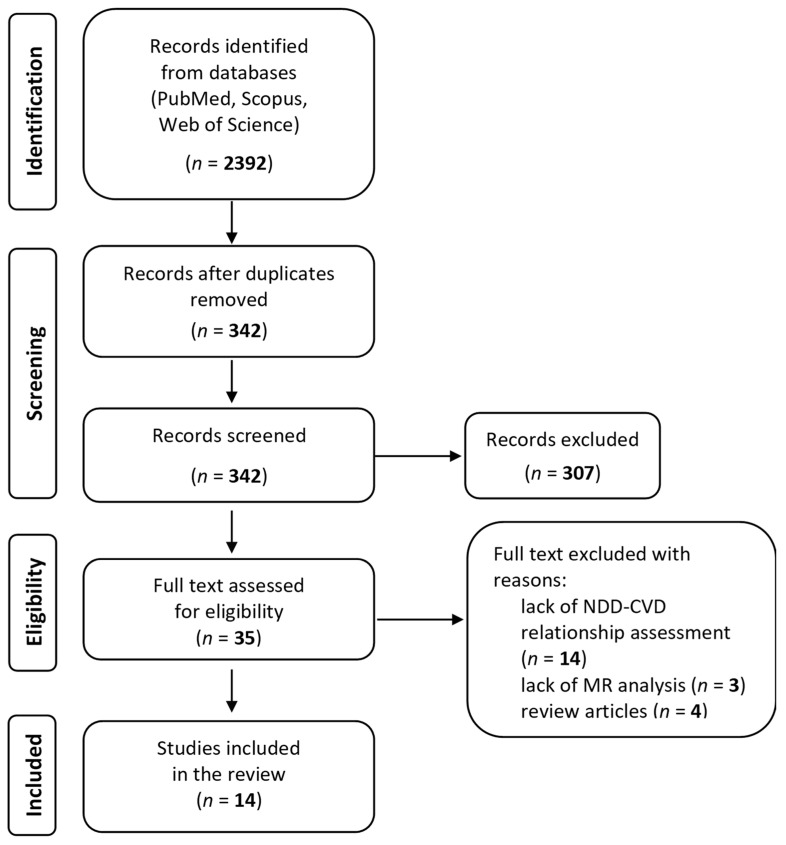
PRISMA flowchart.

The database searches returned a total of 2392 records. After removing duplicates (*n* = 2050), 342 unique records (title and abstract) were left for further full-text review. The literature screening process reduced the selection down to 14 papers, as demonstrated in Figure 1 (PRISMA flowchart). Further details concerning the search results can be found in Supplementary Table S1.

### 3.2. Quality and Bias

A total of 13 out of 14 MR studies included in the meta-analysis [16,33,34,35,36,37,38,39,40,41,42,43,44,45] (Table 1) were evaluated as high quality with a total score exceeding 85% (low risk of bias) in STROBE-MR (Supplementary Tables S2 and S3). In all MR studies, the genetic instruments were strongly associated with the exposure (i.e., *p* < 5 × 10^–8^) (assumption 1—relevance). In most studies, participants were restricted to individuals of European ancestry—an ethnically homogenous population. In some protocols, the genetic association with confounders was assessed using available data or curated databases (e.g., PhenoScanner) [34,41,43], detected outlier SNPs by MR-PRESSO [16,36,37,38,40,41,42,43,44], or employed multivariable MR analysis [33,36,37,44] (assumption 2—independence). The studies used different MR analytic methods that relied on different assumptions, such as the weighted median (majority valid), weighted mode (plurality valid), MR-Egger (instrument strength independent of direct effect) with pleiotropy test, MR-PRESSO, MR-RAPS, Cochran’s Q test, and leave-one-out analysis (assumption 3—exclusion restriction). The inverse variance weighted method (IVW) was chosen as the primary one for MR analysis in 12/14 studies; in one study [39], the MW-IVW method was selected as the primary tool; and in a study by Chen, Peng et al. (2024) [34], the effects of psychiatric disorders on the risk of cardiovascular diseases (CVDs) were assessed using the Wald ratio method. In two studies, power calculation was performed and the power issue was considered [43,44]. All studies reported the sample size for the outcome variables.

### 3.3. Study Characteristics

The most frequently utilized population cohort was the Psychiatric Genomics Consortium (PGC)—for ADHD [33,34,35,38,40,42,44]—and for cardiovascular diseases—the MEGASTROKE consortium [36,40,42], UK Biobank [35,37,45], and CARDIoGRAM*plus*C4D [37,42,44]. The relationship between neurodevelopmental and cardiovascular disorders was analyzed by utilizing MR (IVW, MR-Egger, weighed median, simple mode, or weighted mode), with some studies also performing multivariable MR analysis [33,34,36,37,44]. In 13/14 studies, the risk of cardiovascular diseases in individuals with autism spectrum disorder and/or attention deficit hyperactivity disorder was assessed [33,34,35,36,37,38,39,40,41,42,43,44,45]. Four papers referred to genetic evidence of the causal relationships between cardiovascular diseases and ADHD/ASD [16,33,35,39]. For details, see Table 1 and Supplementary Table S4.

### 3.4. The Relationship Between Neurodevelopmental and Cardiovascular Disorders

**Figure 2:**
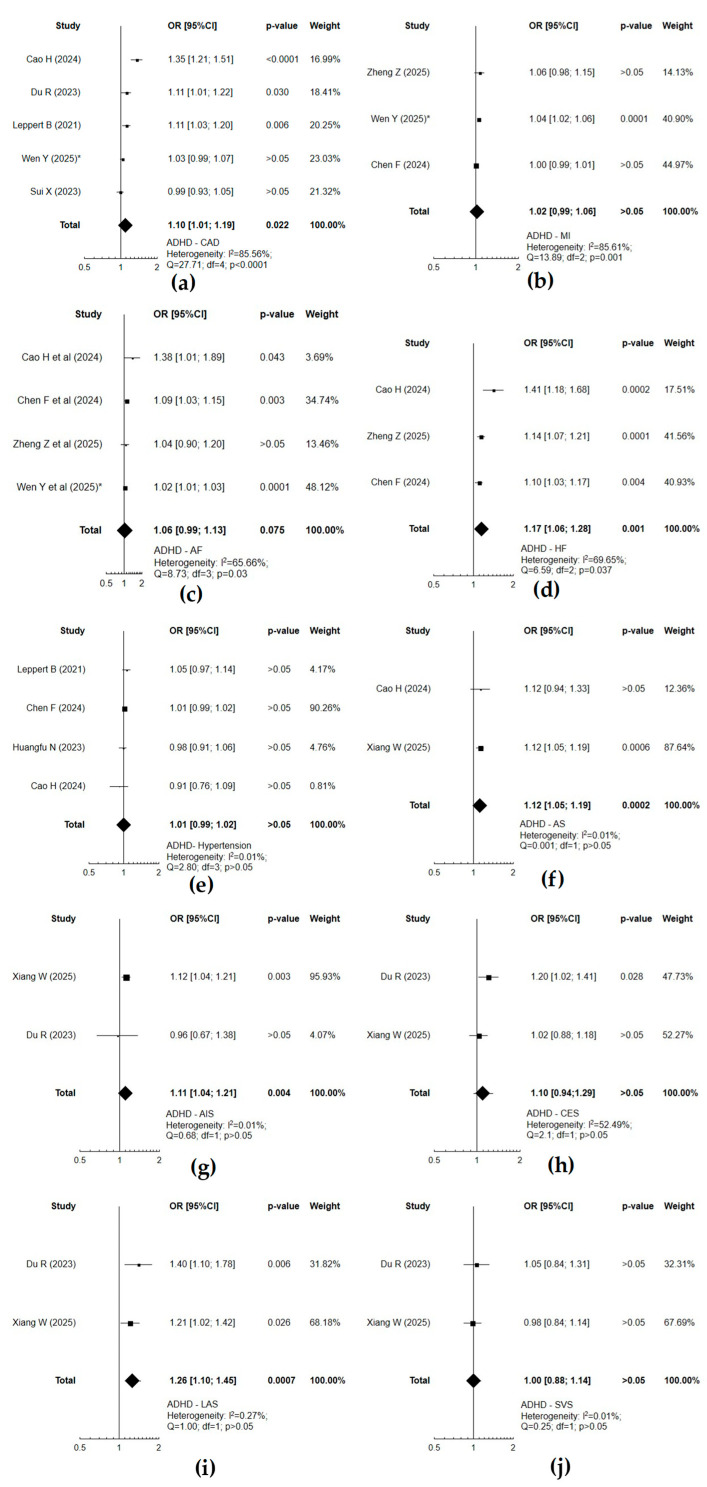
Forest plots of studies that evaluated the causal effect of attention deficit/hyperactivity disorder (ADHD) on cardiovascular diseases using values obtained by the IVW MR method: (**a**) coronary artery disease (CAD); (**b**) myocardial infarction (MI); (**c**) atrial fibrillation (AF); (**d**) heart failure (HF); (**e**) hypertension; (**f**) any stroke (AS); (**g**) any ischemic stroke (AIS); (**h**) cardioembolic stroke (CES); (**i**) large-artery atherosclerotic stroke (LAS); (**j**) small-vessel stroke (SVS) [33,35,36,37,38,39,40,42,45]; *—values obtained by MW-IVW method.

**Figure 3:**
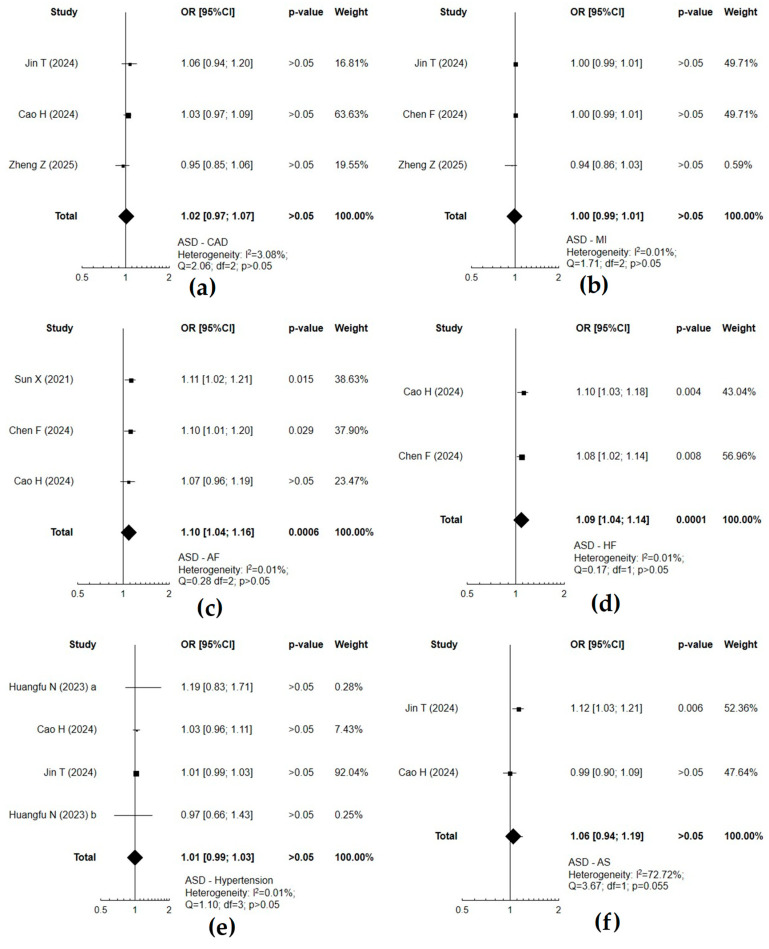
Forest plots of studies that evaluated the causal effect of autism spectrum disorder (ASD) on cardiovascular diseases using values obtained by the IVW MR method: (**a**) coronary artery disease (CAD); (**b**) myocardial infarction (MI); (**c**) atrial fibrillation (AF); (**d**) heart failure (HF); (**e**) hypertension; (**f**) any stroke (AS) [33,35,42,43,44,45]; a—according to FinnGen data source; b—according to UK Biobank.

**Figure 4:**
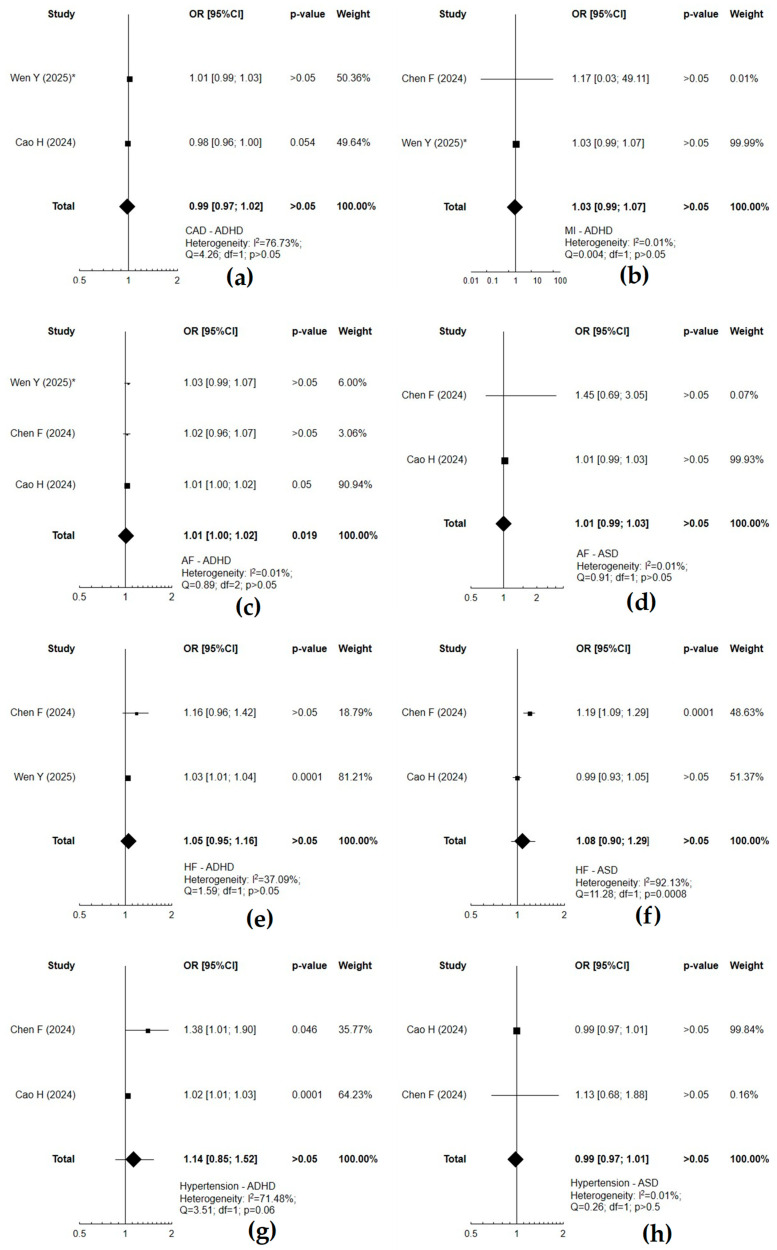
Forest plots of studies that evaluated the causal effect of cardiovascular diseases on attention-deficit/hyperactivity disorder (ADHD) (**a**–**c**,**e**,**g**) and autism spectrum disorder (ASD) (**d**,**f**,**h**) using values obtained by the inverse variance weighted (IVW) Mendelian Randomization (MR) method [33,35,39]. *—values obtained by variance weighted with modified weights (MW-IVW) method. AF—atrial fibrillation; CAD—coronary artery disease; HF—heart failure; MI—myocardial infarction.

When assessing (IVW MR method) the causal effect between neurodevelopmental and cardiovascular disorders, a significant causal effect concerned mainly ADHD and coronary artery disease (*p* = 0.022), heart failure (*p* = 0.001), or stroke and its subtypes (*p* < 0.05) (Figure 2). ASD was associated with an increased risk of atrial fibrillation (*p* = 0.0006) or heart failure (*p* = 0.0001) (Figure 3). Atrial fibrillation was associated with a substantial risk of ADHD (*p* = 0.019) (Figure 4). Forest plots demonstrate the synthesis of results from two or more separate studies. Table 1 and Supplementary Table S4 also present results from single studies, not included in the plots. The analysis of causal effects between neurodevelopmental and cardiovascular disorders assessed using other methods (MR-Egger, weighed median, simple mode, or weighted mode) did not reveal any substantial relationship in the absence of such a significant causality according to IVW.

## 4. Discussion

Numerous systematic reviews and meta-analyses of the observational studies from the last five years suggest that both ADHD and ASD (for references, see Introduction) might increase the risk of CVDs. Moreover, the reverse relationship, i.e., the influence of CVDs on the risk of ADHD or ASD, could also be of importance. However, the underlying mechanisms linking these conditions remain poorly understood. The Mendelian Randomization (MR) provides a robust analytical approach, employing genetic variants as instrumental variables (IVs) to investigate causal links between risk factors and disease outcomes. In this context, the aim of our study was to evaluate the causal effects between those two neurodevelopmental disorders (ADHD, ASD) and cardiovascular diseases in two directions [i.e., ADHD or ASD (exposure) → CVDs (outcome) and CVDs (exposure) → ADHD or ASD (outcome)]. For this purpose, we performed a systematic review and meta-analysis of all MR studies considering the above topic.

The following cardiovascular disease entities were included: congenital heart disease, hypertension, coronary artery disease (coronary heart disease), myocardial infarction, heart failure (congestive heart disease), atrial fibrillation, and stroke (and its subtypes—acute ischemic stroke, cardioembolic stroke, large-artery atherosclerotic stroke, small-vessel stroke). The nomenclature generally stayed in line with the ICD-11 classification; slight differences between individual studies are listed in the footer of Table 1. Disorders that could not be identified in the ICD-11, such as metabolic syndrome [57,58], were not included in the analysis [59]. Moreover, the MR studies covering the role of obesity and diabetes, which can also increase cardiovascular risk, were not a part of this evaluation [60].

The STROBE-MR proposes a set of 20 items intended to facilitate the clear and comprehensive reporting of observational studies using Mendelian Randomization (e.g., one sample, two sample, bidirectional MR studies, one- or two-sample MR studies with multiple exposures or multiple outcomes). While the checklist was not proposed as a formal tool for assessing the quality of MR studies, it is intended to help in designing, performing, and evaluating individual epidemiological reports, for their better accuracy, transparency, and robustness [29]. Most MR studies included in the present meta-analysis were evaluated as high quality with a total score exceeding 85% (low risk of bias). When assessing the bias of individual studies according to the assumptions of instrumental variable (IV) analyses, i.e., relevance, independence, and exclusion restriction, the total scoring for most of MR studies was 5 to 6 (high quality). Most studies provided comprehensive analyses involving several reliable MR approaches and several pleiotropy assessments to avoid possible pleiotropic bias.

The results regarding the causal effects between ADHD, ASD, and cardiovascular diseases are compared with the findings from observational studies from the last five years in Table 2. There is significantly more data from MR available regarding the influence of those two NDDs on CVD outcomes than in the case of the reverse relationships. Moreover, ADHD was more frequently analyzed in all 14 MR studies included in the systematic review and meta-analysis than ASD. The causal association between genetically predicted NDD and CVD outcome (and vice versa) depends on the disease entity analyzed. Genetically predicted ASD was associated with an increased risk for atrial fibrillation and heart failure. ADHD (but not ASD) increased the risk of coronary artery disease and stroke (and its subtypes, with the exception of cardioembolic stroke and small-vessel stroke). On the other hand, both ADHD and ASD did not increase the risk of myocardial infarction and hypertension and vice versa. The lack of evident bidirectional causal relationships for each NDD (i.e., the situations when a given NDD is causally linked to a selected CVD and vice versa) may be at least in part due to the relatively small number of available studies; in some cases, only two non-overlapping comparisons could be identified.

Observational studies are prone to biases like confounding and reverse causation, and MR studies seem to minimize these biases as they leverage genetic variants as instrumental variables to strengthen causal inference. Nevertheless, most results are in line with the findings from the observational studies (for details, see Table 2). Thus, ADHD is connected with a higher risk of coronary artery disease, heart failure, and stroke, but not with hypertension. Similarly, adults with ASD are at a higher risk of heart failure [60]. The relationship between ASD and hypertension, however, seems more complex, as among autistic populations, the risk of this CVD increased [13,64] or remained unchanged [18]. In some cases, even lower blood pressure was observed [19]. In a systematic review by Dhanasekara et al. (2023), ASD was not associated with stroke [18]. To our best knowledge, within the established time frame (2020–2025), the data from observational studies regarding the possible relationship between the two discussed NDDs and atrial fibrillation or myocardial infarction is vastly limited. However, patients with autism were more predisposed to arrhythmias [19]. On the other hand, numerous observational studies demonstrated that congenital heart disease in children increases the risk of ADHD and ASD (for details, see Table 2). However, in the only available MR study, the causality between genetically proxied congenital cardiac malformations and the risk of NDDs was not confirmed [16]. Thus, further efforts are needed to assess the causality between congenital heart disease and NDDs.

Individuals with psychiatric disorders (PDs) are more likely to exhibit one or more CVD-related risk factors; this phenomenon might suggest that the association between both conditions (PDs and CVD) is partially attributed to the clustering of these risk factors [65]. For example, observational studies have proven the causal linkage between ADHD and unhealthy lifestyles including smoking, overweight (obesity), or a sedentary lifestyle, both in adolescents and adulthood [66,67,68]. In line with these findings, the increasing amount of evidence has proven the genetic relationship between a variety of CVD-related risk factors and PDs. For example, Ding et al. (2022) found genetic correlations between obesity and such NDDs as ADHD or ASD [69]. Soler et al. (2023) revealed the genetic liability of ADHD on the frequency of current smoking and a negative effect on past tobacco smoking, in both directions, i.e., for ADHD as exposure and outcome, respectively [70]. Two other MR studies confirmed the genetic liability to ASD as being associated with reduced physical activity [71,72]. Controversially, some studies demonstrate that the risk of certain CVDs can still be increased due to NDDs after controlling for a variety of cardiovascular risk factors [7,34]. Therefore, further investigation is needed to determine whether the mutual genetic relationship between ADHD or ASD and CVDs is related to cardiovascular risk factors. Nevertheless, the substantial proportion of individuals with ADHD or ASD developing CVDs provides a rationale for early interventions within these groups to change lifestyle-related factors such as physical activity, obesity, food consumption, tobacco smoking, or alcohol drinking.

The molecular background for such associations is not fully understood, neither. Hyperactivity of the hypothalamic–pituitary–adrenal (HPA) axis—present in multiple neuropsychiatric diseases—can promote an array of pathophysiologic responses that converge to precipitate CVDs, and therefore, HPA axis regulation has been proposed to be an established risk factor of cardiovascular mortality [73]. Another possible explanation of the casual role of psychological disorders in the etiology of CVDs could include activation of platelet function, acute stress, atherosclerosis, endothelial dysfunction, or increased inflammatory response [34]. These are well-recognized factors that determine the pathophysiology of heart failure, the condition demonstrating a possible genetic linkage with ADHD, as we denoted in the current study. In the opposite direction, increased inflammation (↑ C-reactive protein, Il-6, Il-1β, soluble intercellular adhesion molecule) and oxidative stress, with their negative effects on neurons, could increase the risk of developing NDDs in subjects with CVDs, giving the mechanistic rationale for the observed genetic associations [39,73,74].

Due to the limited number of target studies applied to the final study, expanding the interpretation of the results is difficult. This restricts the ability to draw robust conclusions regarding causality across the full spectrum of CVDs and NDDs. Therefore, these findings should be interpreted with caution, and further high-quality MR studies are needed to strengthen the evidence base. Considering the growing evidence of a relationship between neurodevelopmental disorders such as ADHD and ASD and CVDs, the focus should be on understanding how psychiatric traits, including those of NDDs, contribute to CVD risk. This understanding should then be used to improve mental health treatments and develop preventive strategies for CVDs. In this context, the early detection and effective treatment of NDDs in patients with CVDs are also vital for improving both mental and physical well-being, while also minimizing the risk of exacerbating their heart condition [44].

## 5. Strengths and Limitations

Our study adheres to established STROBE-MR guidelines for quality assessment, ensuring a rigorous and transparent methodology. Most MR studies included in the meta-analysis were evaluated as high quality with a total score exceeding 85% (low risk of bias). The restriction of the population to European ancestry in most studies minimized bias due to racial heterogeneity, but it may limit the generalizability of the results to other populations. In some studies using the FinnGen database, the unique nature of the Finnish population genome may influence the validity of genetic instrumental variants and the generalizability of the results of MR analysis [16,45]. Next, we did not perform a meta-analysis on the MR results for the same exposure–outcome pairs when they used the same data sources. Additional studies are required in more diverse populations. MR studies require a large sample size; thus, one of the most frequent limitations is a lack of power calculations—in 12/14 studies. Such an approach could enable the evaluation of the required effect of exposure on the outcome with at least 80% power, according to the sample size of each outcome. Additionally, the effect of age, sex specificity, or medications for NDDs on the outcomes could be fully explored in the future for a better understanding of the complex relationships.

## 6. Conclusions

In conclusion, our systematic review and meta-analysis of MR studies suggest that genetic predisposition to NDDs such as ADHD and ASD had a substantial effect on cardiovascular diseases. In particular, there was a causal relationship between ADHD and coronary artery disease, heart failure, and several types of stroke, while genetically predicted ASD was correlated with an increased risk of heart failure and atrial fibrillation. The latter condition was also significantly correlated with an increased risk of ADHD. These causal genetic associations seem to confirm previous results from observational studies. On the other hand, hypertension did not show any genetic association with either ADHD or ASD. Moreover, the evidence for bidirectional causal relationships remains insufficient. There was significantly more data available regarding the influence of these NDDs on CVD outcomes than in the case of the reverse relationships. To fill this gap, further research on the possible causal association between genetic predisposition to an individual CVD and NDD is needed. The studies aimed to assess the influence of disease heterogeneity and severity and environmental and lifestyle-related cardiovascular risk factors on those genetically based causal relationships could also be of importance. The relationship between other psychiatric disorders and CVDs, and the underlying mechanisms remain to be elucidated, as well. Policymakers should advocate for the integration of genetic insights into early diagnostic and therapeutic frameworks, emphasizing inclusivity in future research efforts.

## Figures and Tables

**Table 1:** Characteristics of the 14 included Mendelian Randomization (MR) studies on autism and ADHD in relation to cardiovascular diseases.

Study	Ethnicity	Cohort/ Dataset	Type of Analysis	Exposure	Sample Size		Outcome	Sample Size		Odds Ratio ¥ (OR) 95% CI	Conclusion
					Cases	Controls		Cases	Controls		
Liu et al. (2024) [16]	European	PGC (ADHD); iPSYCH-PGC (ASD); FinnGen (CHD)	two-sample MR	ADHD	20,183	35,191	CHD	3459	39,040	0.98 (0.91–1.06)	Children with CHD are at greater risk of developing ADHD
				ASD	18,382	27,969				0.96 (0.84–1.11)	
Cao et al. (2024) [33]	European (mostly)	PGC (ADHD); iPSYCH-PGC (ASD); Nelson et al. (2017) [46] (CAD); Jiang et al. (2019) [47] (hypertension); Shah et al. (2020) [48] (HF); Kurki et al. (2023) [49] (AF, arrhythmias, stroke)	MR and multivariable MR	ADHD	38,691	275,986	Arrhythmias	59,182	204,429	1.15 (0.92–1.44)	ADHD plays significant roles in elevating the chances of CVD
							AF	40,594	168,000	1.38 (1.01–1.88) *	
							CAD	71,602	260,875	1.35 (1.21–1.51) *	
							HF	47,309	930,014	1.41 (1.18–1.7) *	
							Hypertension	122,620	332,683	0.91 (0.76–1.08)	
							Stroke	34,560	249,480	1.12 (0.94–1.34)	
				ASD	18,381	27,969	Arrhythmias	59,182	204,429	1.01 (0.93–1.10)	
							AF	40,594	168,000	1.07 (0.95–1.21)	
							CAD	71,602	260,875	1.03 (0.97–1.09)	
							HF	47,309	930,014	1.1 (1.03–1.18) *	
							Hypertension	122,620	332,683	1.03 (0.96–1.10)	
							Stroke	34,560	249,480	0.99 (0.9–1.09)	
				Arrhythmias	59,182	204,429	ADHD	38,691	275,986	1.02 (1.01–1.04) *	
				AF	40,594	168,000				1.01 (1.00–1.02)	
				CAD	71,602	260,875				0.98 (0.96–1.01)	
				HF	47,309	930,014				1.00 (0.95–1.06)	
				Hypertension	122,620	332,683				1.02 (1.01–1.03) *	
				Stroke	34,560	249,480				1.01 (0.99–1.03)	
				Arrhythmias	59,182	204,429	ASD	18,381	27,969	1.04 (1.00–1.07)	
				AF	40,594	168,000				1.01 (0.99–1.03)	
				CAD	71,602	260,875				0.96 (0.92–1.00)	
				HF	47,309	930,014				0.99 (0.93–1.07)	
				Hypertension	122,620	332,683				0.99 (0.97–1.02)	
				Stroke	34,560	249,480				1.01 (0.97–1.06)	
Chen Y. et al. (2024) [34]	European	PGC (ADHD); iPSYCH-PGC (ASD); Shah et al. (2020) [48] (HF)	univariable and multivariable two-sample MR	ADHD	20,183	35,191	HF	47,309	930,014	1.12 (1.04–1.2) *	ADHD and ASD may have a causal relationship with an increased risk of HF
				ASD	18,382	27,969	HF	47,309	930,014	1.29 (1.07–1.56) *^,&^	
Chen F. et al. (2024) [35]	European	PGC (ADHD); iPSYCH-PGC (ASD); UK Biobank (hypertension); Roselli et al. (2018) [50] (AF); Nelson et al. (2017) [46] (CAD); Levin et al. (2022) [51] (HF); Hartiala et al. (2021) [52] (MI)	bidirectional MR	ADHD	38,691	186,843	AF	65,446	588,190	1.088 (1.026–1.153) *	Further studies are needed for the shared genetic etiology
							CAD	60,801	123,504	1.187 (1.087–1.297) *	
							Cardiomyopathy	361,194		1.000 (0.999–1.001)	
							HF	115,150	1,550,331	1.097 (1.032–1.165) *	
							Hypertension	361,194		1.007 (0.994–1.021)	
							MI	61,000	578,000	1.005 (1.001–1.009) *	
				ASD	18,381	27,969	AF	65,446	588,190	1.099 (1.011–1.195) *	
							CAD	60,801	123,504	0.999 (0.994–1.004)	
							Cardiomyopathy	361,194		1.000 (0.999–1.006)	
							HF	115,150	1,550,331	1.077 (1.019–1.139) *	
							Hypertension	361,194		1.001 (0.986–1.015)	
							MI	61,000	578,000	0.999 (0.995–1.003)	
				AF	65,446	588,190	ADHD	38,691	186,843	1.017 (0.964–1.074)	
				CAD	60,801	123,504				0.973 (0.911–1.039)	
				HF	115,150	1,550,331				1.165 (0.956–1.419)	
				Hypertension	361,194					1.385 (1.006–1.907) *	
				MI	61,000	578,000				1.169 (0.028–49.112)	
				AF	65,446	588,190	ASD	18,381	27,969	1.458 (0.696–3.056)	
				CAD	60,801	123,504				0.958 (0.899–1.021)	
				HF	115,150	1,550,331				1.188 (1.091–1.294) *	
				Hypertension	361,194					1.134 (0.676–1.904)	
				MI	61,000	578,000				1.241 (0.011–141.915)	
Du et al. (2023) [36]	European	Demontis et al. (2019) [22] (ADHD); MEGASTROKE (CVDs)	two-sample MR	ADHD	19,099	34,194	AIS	40,585	406,111	0.96 (0.67–1.38)	Genetic predisposition to ADHD was associated with an enhanced risk of AIS, particularly LAS
							LAS	40,585	406,111	1.4 (1.10–1.76) *	
							CES	40,585	406,111	1.20 (1.02–1.41) *	
							SVS	40,585	406,111	1.05 (0.84–1.31)	
							CAD	40,585	406,111	1.11 (1.01–1.22) *	
Leppert et al. (2021) [37]	European	Demontis et al. (2019) [22] (ADHD); CARDIoGRAM*plus*C4D (CAD, MI); UK Biobank (hypertension)	bidirectional two-sample MR	ADHD	19,099	34,194	CAD	60,801	123,504	1.11 (1.03–1.19) *	The findings support a causal relationship between ADHD and CAD
							MI	43,676	128,199	1.06 (0.97–1.16)	
							Hypertension	87,690	249,469	1.05 (0.97–1.13)	
Sui et al. (2023) [38]	European	PGC (ADHD); van der Harst and Verweij (2018) [53] (CAD); Shah et al. (2020) [48] (HF); Roselli et al. (2018) [50] (AF); Malik et al. (2018) [54] (AIS)	two-sample MR	ADHD	20,183	35,191	CAD	122,733	424,528	0.99 (0.93–1.06)	ADHD is associated with an increased risk of HF, AF, and IS
							AF	55,114	482,295	1.08 (1.02–1.15) *	
							HF	47,309	930,014	1.12 (1.04–1.20) *	
							IS	440,328		1.15 (1.05–1.25) *	
Wen et al. (2025) [39]	European	PGC (ADHD); CARDIo-GRAM (CAD, MI); Shah et al. (2020) [48] (HF); Nielsen et al. (2018) [55] (AF)	bidirectional two-sample MR	ADHD	38,691	186,843	AF	60,620	970,216	1.011 (1.009–1.030) *^,MW^	There are bidirectional causal relationships between HF and ADHD
							CAD	22,233	64,762	1.032 (0.994–1.070) ^MW^	
							HF	47,309	930,014	1.027 (1.014–1.039) *^,MW^	
							MI	42,335	78,240	1.039 (1.025–1.051) *^,MW^	
				AF	60,620	970,216	ADHD	38,691	186,843	1.029 (0.991–1.067) ^MW^	
				CAD	22,233	64,762				1.010 (0.985–1.035) ^MW^	
				HF	47,309	930,014				1.025 (1.013–1.038) *	
				MI	42,335	78,240				1.032 (0.991–1.073) ^MW^	
Xiang et al. (2025) [40]	European	PGC (ADHD); MEGASTROKE (CVDs)	bidirectional two-sample MR	ADHD	38,691	186,843	AS	40,585		1.118 (1.047–1.195) *	Genetically predicted ADHD increases the risk of LAS; ASD but not ADHD is causally linked to CVD.
							AIS	34,217		1.118 (1.035–1.206) *	
							LAS	4373		1.206 (1.023–1.422) *	
							CES	7197		1.023 (0.876–1.195)	
							SVS	5386		0.980 (0.843–1.138)	
Yu et al. (2024) [41]	European	UK Biobank (ADHD, ASD); FinnGen (CVDs)	MR	ADHD	55,374		CVD	377,277		1.02 (0.99–1.06)	ASD but not ADHD is causally linked to CVD
				ASD	46,351					1.05 (1.00–1.09)	
Zheng and Cai (2025) [42]	European	PGC (ADHD); iPSYCH-PGC (ASD); CARDIoGRAM*plus*C4D (MI, CAD); HERMES (HF); MEGASTROKE (LAS, CES, SVS); Nielsen et al. (2018) [55] (AF)	two-sample MR	ADHD	20,183	35,181	MI	43,676	128,188	1.062 (0.971–1.162)	Cardiovascular monitoring in individuals with ADHD or ASD is crucial to prevent associated risk factors
							AF	60,620	970,216	1.042 (0.896–1.101)	
							HF	47,309	930,014	1.139 (1.065–1.218) *	
							CAD	60,801	123,304	1.115 (1.029–1.209) *	
							LAS	7193	406,111	1.345 (1.092–1.656) *	
							CES	4373	406,111	1.144 (0.973–1.345)	
							SVS	5386	406,111	1.088 (0.896–1.322)	
				ASD	18,381	27,969	MI	43,676	128,188	0.939 (0.857–1.029)	
							AF	60,620	970,216	1.089 (1.026–1.155) *	
							HF	47,309	930,014	1.112 (1.035–1.194) *	
							CAD	60,801	123,304	0.953 (0.849–1.069)	
							LAS	7193	406,111	1.13 (0.911–1.403) *	
							CES	4373	406,111	1.038 (0.877–1.228)	
							SVS	5386	406,111	1.084 (0.888–1.324)	
Jin et al. (2024) [43]	European	iPSYCH-PGC (ASD); Nielsen et al. (2018) [55] (AF); Malik et al. (2018) [54] (AS, AIS, LAS, CES, SVS); van der Harst and Verweij (2018) [53] (CAD); Dönertaş et al. (2021) [56] (MI, hypertension)	two-sample MR	ASD	18,382	27,969	AF	60,620	970,216	1.082 (1.0019–1.1684) *	Causal relationships between ASD and AS, IS, LAS, and HF
							HF	47,309	930,014	1.102 (1.001–1.213) *	
							CAD	122,733	424,528	1.059 (0.943–1.189)	
							MI	11,081	473,517	1.001 (0.9980–1.004)	
							Hypertension	129,909	354,689	1.01 (0.99–1.02)	
							AS	40,585	406,111	1.118 (1.032–1.214) *	
							IS	34,217	406,111	1.116 (1.024–1.216) *	
							LAS	47,309	406,111	1.290 (1.039–1.601) *	
							CES	122,733	406,111	0.994 (0.830–1.191)	
							SVS	11,081	406,111	1.205 (0.975–1.488)	
Sun et al. (2021) [44]	European (mostly)	iPSYCH-PGC (ASD); CARDIoGRAM*plus*C4D (CAD, MI); HERMES (HF); Nielsen et al. (2018) [55] (AF)	two-sample MR	ASD	18,381	27,969	CAD	60,801	123,504	0.997 (0.897–1.106)	Genetic predisposition to ASD was associated with a higher risk of AF and HF
							MI			0.993 (0.883–1.117)	
							AF	60,620	970,216	1.109 (1.023–1.201) *	
							HF	47,309	930,014	1.138 (1.036–1.251) *	
Huangfu et al. (2023) [45]	European	PGC (ADHD); iPSYCH-PGC (ASD); FinnGen and UK Biobank (hypertension)	two-sample MR	ADHD	20,183	35,191	Hypertension	42,857	162,837	0.98 (0.91–1.07)	No links were identified between genetic predisposition to ASD or ADHD and the risk of hypertension
								54,358	408,652	1.10 (1.00–1.19)	
				ASD	18,381	27,969		42,857	162,837	1.19 (0.83–1.71)	
								54,358	408,652	0.97 (0.66–1.42)	

ADHD, attention-deficit hyperactivity disorder; AF, atrial fibrillation; AS, any stroke; (A)IS, (any) ischemic stroke; ASD, autism spectrum disorder; CAD, coronary artery disease; CARDIoGRAM*plus*C4D, Coronary Artery Disease Genome-Wide Replication and Meta-analysis plus the Coronary Artery Disease Genetics Consortium; CES, cardioembolic stroke; CHD, congenital heart disease; CVDs, cardiovascular diseases; HERMES, Heart Failure Molecular Epidemiology for Therapeutic Targets Consortium; HF, heart failure; IS, ischemic stroke; LAS, large-artery atherosclerotic stroke; MI, myocardial infarction; MR, Mendelian Randomization; PGC, Psychiatric Genomics Consortium; SVS, small-vessel stroke. ¥—values obtained by MW-IVW method; ^&^—values obtained by Wald ratio method; ^MW^—values obtained by MW-IVW method; *—*p* < 0.05. For further statistical analyses, the results for “any stroke” [40,43] were combined with those for “stroke” [33], and “any ischemic stroke” [36,40] with “ischemic stroke” [38,43]. The terms “coronary heart disease” [42] and “coronary artery disease” [33,35,37,38,39,43,44] were treated as referring to the same medical condition. The results from studies assessing the relationship between ADHD and/or ASD and CVDs (in general) were not included in the detailed meta-analyses.

**Table 2:** Summary of the findings from the present systematic review and meta-analysis of Mendelian Randomization (MR) studies (Figure 2, Figure 3 and Figure 4 and Supplementary Table S4) and their relation to meta-analyses and systematic reviews of observational studies from the last five years.

Exposure(s)	Outcomes	MR	Exposure(s)	Outcomes	MR	Observational Studies
ADHD	CAD	+	CAD	ADHD	−	ADHD increases the risk of CAD [7]
	MI	−	MI		−	no data
	AF	−	AF		+	no data
	HF	+	HF		−	ADHD increases the risk of HF [61]
	CHD	n.d.	CHD		+ ^1^	CHD in children increases the risk of ADHD [5,14,15,16,17,62,63]
	hypertension	−	hypertension		−	ADHD is associated (but not significantly) with a higher risk of hypertension [5]
	AS	+	AS		n.d.	ADHD increases the risk of stroke [7], including ischemic [12] and hemorrhagic [8]
	AIS	+	AIS		n.d.	
	CES	−	CES		n.d.	
	LAS	+	LAS		n.d.	
	SVS	−	SVS		n.d.	
ASD	CAD	−	CAD	ASD	n.d.	heart diseases have greater odds in older autistic adults [11]
	MI	−	MI		− ^1^	no data
	AF	+	AF		−	patients with ASD are more predisposed to arrhythmias; no data regarding AF [19]
	arrythmias	− ^1^	arrythmias		− ^1^	
	HF	+	HF		−	adults with ASD are at a higher risk of HF [60]
	CHD	n.d.	CHD		− ^1^	children with CHD have an increased risk of ASD [5,14,15,16,17,62,63]
	hypertension	−	hypertension		−	within ASD populations: (1) higher prevalence of hypertension [64] or its modest increase [13]; (2) no significant increase in its risk [18]; or (3) lower blood pressure [19]
	AS	−	AS		n.d.	ASD is not associated with an increased risk of stroke [18]
	AIS	n.d.	AIS		n.d.	
	CES	n.d.	CES		n.d.	
	LAS	n.d.	LAS		n.d.	
	SVS	n.d.	SVS		n.d.	

^1^ only 1 study. ADHD, attention-deficit hyperactivity disorder; AF, atrial fibrillation; AS, any stroke; AIS, any ischemic stroke; ASD, autism spectrum disorder; CAD, coronary artery disease; CES, cardioembolic stroke; CHD, congenital heart disease; HF, heart failure; IS, ischemic stroke; LAS, large-artery atherosclerotic stroke; MI, myocardial infarction; MR, Mendelian Randomization; n.d., not determined; SVS, small-vessel stroke; +, causality confirmed; −, causality not confirmed.

## Data Availability

The original contributions presented in this study are included in the article/Supplementary Materials. Further inquiries can be directed to the corresponding author.

## Supplementary Materials

The following supporting information can be downloaded at: https://www.mdpi.com/article/10.3390/cells14151180/s1, Table S1: Search results; Table S2: Quality assessment according to STROBE-MR guidelines; Table S3: Bias assessment results; Table S4: Data extraction results.

## Abbreviations

The following abbreviations are used in this manuscript:

ADHD	attention deficit hyperactivity disorder
AF	atrial fibrillation
AIS	any ischemic stroke
AS	any stroke
ASD	autism spectrum disorder
CAD	coronary artery disease
CES	cardioembolic stroke
CVD	cardiovascular disease
GWASs	genome-wide association studies
HF	heart failure
IVW	inverse variance weighted
LAS	large-artery atherosclerotic stroke
MI	myocardial infarction
MR	Mendelian randomization
MW-IVW	variance weighted with modified weights
PDs	psychiatric disorders
PGC	the Psychiatric Genomics Consortium
SNPs	single nucleotide polymorphisms
SSGAC	Social Science Genetics Association Consortium
SVS	small-vessel stroke
