# Supplementary material for: Evaluating the Causal Effects of ADHD and Autism on Cardiovascular Diseases and Vice Versa: A Systematic Review and Meta-Analysis of Mendelian Randomization Studies
Source: Cells. 2025 Jul 31;14(15):1180. doi: 10.3390/cells14151180 (PMC12345925; doi:10.3390/cells14151180)
Supplement: Supplementary file 1 [file cells-14-01180-s001.zip › Supplementary Table S1.pdf]

Table S1. Search results.

Database	PubMed	Scopus	Web of Science
"mendelian randomization" AND			
"ADHD" OR	213	182	182
"attention-deficit hyperactivity disorder"	188	276	204
"mendelian randomization" AND "ADHD" AND			
"cardiovascular disease" OR	16	5	5
"CVD" OR	4	3	3
"congenital heart disease" OR	2	18	1
"CHD" OR	1	1	2
"hypertension" OR	2	2	1
"coronary heart disease" OR	1	1	2
"myocardial infarction" OR	2	1	1
"MI" OR	14	2	6
"arrythmia" OR	1	1	0
"atrial fibrillation" OR	3	3	2
"AF" OR	1	1	1
"heart failure" OR	7	4	4
"HF" OR	1	1	4
"stroke" OR	8	6	6
"ischemic stroke" OR	3	3	1
"large-artery stroke" OR	2	2	2
"small-vessel stroke" OR	0	0	0
"cardioembolic stroke" OR	0	1	0
"mendelian randomization" AND "attention-deficit hyperactivity disorder" AND			
"cardiovascular disease" OR	8	13	8
"CVD" OR	4	4	4

"congenital heart disease" OR	2	24	3
"CHD" OR	1	1	2
"hypertension" OR	2	9	2
"coronary heart disease" OR	1	1	3
"myocardial infarction" OR	2	2	2
"MI" OR	12	2	4
"arrythmia" OR	1	0	0
"atrial fibrillation" OR	3	5	3
"AF" OR	12	1	3
"heart failure" OR	7	9	7
"HF" OR	1	1	3
"stroke" OR	7	11	14
"ischemic stroke" OR	3	6	2
"large-artery stroke" OR	2	4	1
"small-vessel stroke" OR	0	1	0
"cardioembolic stroke" OR	3	2	0
"mendelian randomization" AND			
"autism" OR	145	204	166
"autism spectrum disorder" OR	116	133	121
"ASD"	83	93	86
"mendelian randomization" AND "autism" AND			
"cardiovascular disease" OR	3	9	6
"CVD" OR	3	5	3
"congenital heart disease" OR	3	2	1
"CHD" OR	1	1	1
"hypertension" OR	1	6	2
"coronary heart disease" OR	1	1	3
"myocardial infarction" OR	2	3	1
"MI" OR	4	1	6
"arrythmia" OR	0	0	0
"atrial fibrillation" OR	1	4	3
"AF" OR	2	2	2

"heart failure" OR	5	6	5
"HF" OR	2	2	3
"stroke" OR	5	7	11
"ischemic stroke" OR	1	4	2
"large-artery stroke" OR	1	2	1
"small-vessel stroke" OR	0	1	0
"cardioembolic stroke" OR	0	3	0
"mendelian randomization" AND "autism spectrum disorder" AND			
"cardiovascular disease" OR	3	5	6
"CVD" OR	3	3	3
"congenital heart disease" OR	0	0	1
"CHD" OR	4	0	1
"hypertension" OR	1	6	2
"coronary heart disease" OR	1	1	2
"myocardial infarction" OR	1	3	1
"MI" OR	4	1	3
"arrythmia" OR	0	0	0
"atrial fibrillation" OR	2	4	3
"AF" OR	2	2	2
"heart failure" OR	5	6	5
"HF" OR	2	2	2
"stroke" OR	4	7	6
"ischemic stroke" OR	1	4	1
"large-artery stroke" OR	1	2	1
"small-vessel stroke" OR	0	1	0
"cardioembolic stroke" OR	0	3	0
"mendelian randomization" AND "ASD" AND			
"cardiovascular disease" OR	3	5	4
"CVD" OR	3	3	3
"congenital heart disease" OR	0	0	0
"CHD" OR	0	0	0
"hypertension" OR	1	3	1

"coronary heart disease" OR	1	1	0
"myocardial infarction" OR	1	1	1
"MI" OR	4	1	2
"arrythmia" OR	0	0	0
"atrial fibrillation" OR	2	3	3
"AF" OR	2	2	2
"heart failure" OR	4	5	4
"HF" OR	2	2	2
"stroke" OR	2	2	4
"ischemic stroke" OR	1	1	1
"large-artery stroke" OR	1	1	1
"small-vessel stroke" OR	0	0	0
"cardioembolic stroke" OR	0	0	0
