# Supplementary material for: Evaluating the Causal Effects of ADHD and Autism on Cardiovascular Diseases and Vice Versa: A Systematic Review and Meta-Analysis of Mendelian Randomization Studies
Source: Cells. 2025 Jul 31;14(15):1180. doi: 10.3390/cells14151180 (PMC12345925; doi:10.3390/cells14151180)
Supplement: Supplementary file 1 [file cells-14-01180-s001.zip › Supplementary Table S2.pdf]

Table S2. Quality assessment according to STROBE-MR guidelines.

				Title and Abstract	Introduction		Methods						Results				Discussion				Other information			
Title	First author	Year	PMID	Title and Abstract	Background	Objectives	Study design and data sources	Assumptions	Statistical methods: main analysis	Assessment of assumptions	Sensitivity analyses	Software and pre-registration	Descriptive data	Main results	Assessment of assumptions	Sensitivity and additional	Key results	Limitations	Interpretation	Generalizability	Funding	Data and data sharing	Conflicts of interest	Total score out of 20 (%)
Bidirectional associations between mental disorders, antidepressants and cardiovascular disease.	Cao H	2024	38490691	1	1	1	1	0,5	0,5	0,5	1	0,5	1	1	0,5	1	1	1	1	1	1	1	1	87,5
The brain-heart axis: Integrative analysis of the shared genetic etiology between neuropsychiatric disorders and cardiovascular disease.	Chen F	2024	38518856	1	1	1	1	0,5	1	1	0,5	0,5	1	1	0,5	1	1	1	1	1	1	1	1	90,0
The effects of psychiatric disorders on the risk of chronic heart failure: a univariable and multivariable Mendelian randomization study.	Chen Y	2024	38299073	1	1	1	1	1	1	1	1	0,5	1	1	1	0,5	1	1	1	1	1	1	1	95,0
Attention-deficit/hyperactivity disorder and ischemic stroke: A Mendelian randomization study.	Du R	2023	35670701	1	1	1	1	1	1	1	1	0,5	1	1	1	1	1	1	1	1	1	1	1	97,5

Genetic liability to mental disorders in relation to the risk of hypertension.	Huangfu N	2023	36923957	1	1	1	1	1	1	1	1	0,5	1	1	1	1	1	1	1	1	1	1	1	97,5
Genetically identified mediators associated with increased risk of stroke and cardiovascular disease in individuals with autism spectrum disorder.	Jin T	2024	38640796	1	1	1	1	1	1	1	1	0,5	1	1	1	1	1	1	1	0,5	1	1	1	95,0
The Effect of Attention Deficit/Hyperactivity Disorder on Physical Health Outcomes: A 2-Sample Mendelian Randomization Study.	Leppert B	2021	33324987	1	1	1	1	1	1	1	1	0,5	1	1	1	1	1	1	1	1	1	0	1	92,5
Association of congenital heart disease and neurodevelopmental disorders: an observational and Mendelian randomization study	Liu ZY	2024	38589916	1	1	1	1	0,5	1	0,5	1	0,5	1	1	0,5	1	1	1	1	1	1	1	1	90,0
Psychiatric disorders and cardiovascular diseases: A mendelian randomization study.	Sui X	2023	37842613	1	1	1	1	1	1	1	1	0,5	1	1	1	1	1	1	1	1	0	1	1	92,5
Association of Autism Spectrum Disorder, Neuroticism, and Subjective Well-Being With Cardiovascular Diseases: A Two-Sample Mendelian Randomization Study.	Sun X	2021	34179139	1	1	1	1	0,5	1	1	1	0,5	1	1	1	1	1	1	1	1	1	1	1	95,0
Genetic evidence of the causal relationships between psychiatric disorders and cardiovascular diseases.	Wen Y	2025	39752762	1	1	1	1	1	1	1	1	0,5	1	1	1	1	1	1	1	1	1	1	1	97,5
Causal association between mental disorders and cerebrovascular diseases: Evidence from Mendelian randomization study.	Xiang W	2025	39271072	1	1	1	1	1	1	1	1	0,5	1	1	1	1	1	1	1	1	1	1	1	97,5
A Mendelian randomization study of the effect of mental disorders on cardiovascular disease.	Yu Y	2024	38887450	1	1	1	1	1	1	1	1	0,5	1	1	1	1	1	1	1	1	1	1	1	97,5
Causality Between ADHD, ASD, and CVDs: A Two-Step, Two-Sample Mendelian Randomization Investigation.	Zheng Z	2025	39402923	1	1	1	1	1	1	1	0,5	0,5	1	1	1	1	1	1	1	1	1	1	1	95,0

Each item is scored between 0 and 1 for each criterion to yield a total score
