# Supplementary material for: Evaluating the Causal Effects of ADHD and Autism on Cardiovascular Diseases and Vice Versa: A Systematic Review and Meta-Analysis of Mendelian Randomization Studies
Source: Cells. 2025 Jul 31;14(15):1180. doi: 10.3390/cells14151180 (PMC12345925; doi:10.3390/cells14151180)
Supplement: Supplementary file 1 [file cells-14-01180-s001.zip › Supplementary Table S3.pdf]

Table S3. Bias assessment results.

First author	Year	Title	PMID	Assumption 1 (Relevance)	Assumption 2 (Independence)	Assumption 3 (Exclusion restriction)	Other considerations	Total scores (Quality of study)
**Liu ZY**	**2024**	Association of congenital heart disease and neurodevelopmental disorders: an observational and Mendelian randomization study	38589916	**Satisfied (score = 2)**<br><br>1) Genetic instruments were at genome-wide significance with the exposure ($p < 5 \times 10^{-8}$) (CAD and ADHD);<br>2) The F-statistics of genetic instruments were greater than 10.	**Satisfied (score = 2)**<br><br>1) MR-PRESSO method was performed to detect and remove outliers;<br>2) Study participants were restricted to individuals of European ancestry.	**Satisfied (score = 1)**<br><br>1) The study conducted different MR analytic methods: IVW-MR, weighted median, and MR-Egger, Cochran's Q test (heterogeneity), the intercept of MR-Egger (pleiotropy), the leave-one-out analysis (sensitivity analysis) were used to assess potential biases.	Power calculation was not performed and power issue was considered.	**5 (High quality)**
**Cao H**	**2024**	Bidirectional associations between mental disorders, antidepressants and cardiovascular disease.	38490691	**Satisfied (score = 1)**<br><br>1) Genetic instruments were at genome-wide significance with the	**Satisfied (score = 2)**<br><br>1) Study examined the genetic association with confounders using multivariable MR analysis;	**Satisfied (score = 1)**<br><br>1) The study conducted different MR analytic methods: inverse variance weighted (IVW), MR-Egger, weighted median model; MR-Egger intercept was used to assess the presence of average directional pleiotropy; the heterogeneities	Power calculation was not performed and power issue was not considered.	**4 (Low-to-moderate quality)**

				exposure (p < 5 × 10⁻⁸).	2) Study participants are restricted to individuals of European ancestry.	were analysed by I^2 statistics and Cochran's Q test.		
**Chen F**	**2024**	The brain-heart axis: Integrative analysis of the shared genetic etiology between neuropsychiatric disorders and cardiovascular disease.	38518856	**Satisfied (score = 2)**<br><br>1) Genetic instruments were at genome-wide significance with the exposure ($p < 5 \times 10^{-8}$);<br>2) The F-statistics of genetic instruments were greater than 10.	**Satisfied (score = 2)**<br><br>1) SNPs potentially associated with the outcome phenotypes were excluded by the PhenoScanner check;<br>2) Study participants were restricted to individuals of European ancestry.	**Satisfied (score = 1)**<br><br>1) The study conducted different MR analytic methods: inverse variance weighted (IVW), MR-Egger, and weighted median; the heterogeneities were analysed by Cochran's Q test and leave-one-out analyses, and pleiotropy was tested by the MR-Egger intercept test, MR-PRESSO global test, and funnel plots.	Power calculation was not performed and power issue was not considered.	**5 (High quality)**
**Chen Y**	**2024**	The effects of psychiatric disorders on the risk of chronic heart failure: a univariable and multivariable Mendelian randomization study.	38299073	**Satisfied (score = 2)**<br><br>1) Genetic instruments were at genome-wide significance with the exposure ($p < 5 \times 10^{-8}$); 2) The F-	**Satisfied (score = 2)**<br><br>1) Study examined the genetic association with confounders using multivariable MR analysis;<br>2) Study participants were	**Satisfied (score = 1)**<br><br>1) The multiplicative inverse variance-weighted (IVW) method was used in the univariable MR analysis; the heterogeneities were analysed by Cochran's Q test and leave-one-out analyses, and pleiotropy was tested by the MR-Egger intercept test.	Power calculation was not performed and power issue was not considered.	**5 (High quality)**

				statistics of genetic instruments were greater than 10.	restricted to individuals of European ancestry.			
**Du R**	**2023**	Attention-deficit/hyperactivity disorder and ischemic stroke: A Mendelian randomization study.	35670701	**Satisfied (score = 2)**<br><br>1) Genetic instruments were at genome-wide significance with the exposure ($p < 5 \times 10^{-8}$);<br>2) The F-statistics of genetic instruments were greater than 10.	**Satisfied (score = 2)**<br><br>1) Study examined the genetic association with confounders using multivariable MR analysis; MR-PRESSO method was performed to detect and remove outliers;<br>2) Study participants were restricted to individuals of European ancestry.	**Satisfied (score = 1)**<br><br>1) The study conducted different MR analytic methods: inverse variance weighted (IVW), MR Egger, and MR-RAPS; the leave-one-out analysis or MR-PRESSO outlier test was conducted to detect potential outliers;<br>2) The heterogeneity and pleiotropy were assessed by Cochran Q statistics, MR-Egger intercept, and MR-PRESSO.	Power calculation was not performed and power issue was not considered.	**5 (High quality)**
**Huangfu N**	**2023**	Genetic liability to mental disorders in relation to the risk of hypertension.	36923957	**Satisfied (score = 2)**<br><br>1) Genetic instruments were at genome-wide significance with the	**Satisfied (score = 2)**<br><br>1) MR-PRESSO method was performed to detect and remove outliers;<br>2) Study participants were restricted to	**Satisfied (score = 1)**<br><br>1) The multiplicative inverse variance-weighted (IVW) method was used in the univariable MR analysis; the heterogeneities were analysed by Cochran's Q test, and pleiotropy was tested by the MR-Egger intercept test;	Power calculation was not performed and power issue was considered.	**5 (High quality)**

				exposure (p < 5 × 10−8);<br>2) The F-statistics of genetic instruments were greater than 10.	individuals of European ancestry.	2) For two cohorts (each), the random-effects inverse-variance weighted (IVW) method was used to assess the associations of mental health disorders with hypertension.		
**Jin T**	**2024**	Genetically identified mediators associated with increased risk of stroke and cardiovascular disease in individuals with autism spectrum disorder.	38640796	**Satisfied (score = 2)**<br><br>1) Genetic instruments were at genome-wide significance with the exposure ($p < 5 \times 10^{-8}$);<br>2) The F-statistics of genetic instruments were greater than 10.	**Satisfied (score = 2)**<br><br>1) SNPs associated with confounding factors were excluded using PhenoScanner V2; MR-PRESSO method was performed to detect and remove outliers;<br>2) Study participants were restricted to individuals of European ancestry.	**Satisfied (score = 1)**<br><br>1) The study conducted different MR analytic methods: inverse variance weighted (IVW), MR-Egger, and weighted median, multiple sensitivity analyses to assess the potential biases: Cochran's Q test (heterogeneity), the intercept of MR-Egger (pleiotropy), the leave-one-out analysis (sensitivity analysis) and funel plot.	Power value of the MR was calculated using the platform available at https://shiny.cnsgenomics.com/mRnd and power issue was considered.	**5 (High quality)**
**Leppert B**	**2021**	The Effect of Attention Deficit/Hyperactivity Disorder on Physical Health Outcomes: A 2-Sample Mendelian Randomization Study.	33324987	**Satisfied (score = 2)**<br><br>1) Genetic instruments were at genome-wide significance with the exposure ($p < 5 \times 10^{-8}$) (CAD and ADHD);	**Satisfied (score = 2)**<br><br>1) Study examined the genetic association with confounders using multivariable MR analysis; MR-PRESSO method was performed to	**Satisfied (score = 1)**<br><br>1) The study conducted different MR analytic methods: inverse variance weighted (IVW), MR-Egger, and weighted median, Cochran's Q test (heterogeneity), the intercept of MR-Egger (pleiotropy), the leave-one-out analysis (sensitivity analysis) were used to assess potential biases.	Power calculation was not performed and power issue was considered.	**5 (High quality)**

				2) The F-statistics of genetic instruments were greater than 10.	detect and remove outliers; 2) Study participants were restricted to individuals of European ancestry.			
**Sui X**	**2023**	Psychiatric disorders and cardiovascular diseases: A mendelian randomization study.	37842613	**Satisfied (score = 2)**<br><br>1) Genetic instruments were at genome-wide significance with the exposure ($p < 5 \times 10^{-8}$); 2) The F-statistics of genetic instruments were greater than 10.	**Satisfied (score = 2)**<br><br>1) MR-PRESSO test was used to detect abnormal SNPs affecting the results and re-calibrating the results after removing the outliers; 2) Study participants were restricted to individuals of European ancestry.	**Satisfied (score = 1)**<br><br>1) The study conducted different MR analytic methods: inverse variance weighted (IVW), MR-Egger, and weighted median, and the reliability of the results was increased using sensitivity analyses such as MR-Egger, Cochrane's Q test, MR-PRESSO and leave-one-out.	Power calculation was not performed and power issue was not considered.	**5 (High quality)**
**Sun X**	**2021**	Association of Autism Spectrum Disorder, Neuroticism, and Subjective Well-Being With Cardiovascular Diseases: A Two-Sample Mendelian Randomization Study.	34179139	**Satisfied (score = 2)**<br><br>1) Genetic instruments were at genome-wide significance with the exposure ($p < 5 \times 10^{-8}$); 2) The F-statistics of genetic	**Satisfied (score = 2)**<br><br>1) MR-PRESSO test was used to detect abnormal SNPs affecting the results and re-calibrating the results after removing the outliers; multivariable MR was performed to evaluate the	**Satisfied (score = 1)**<br><br>1) The study conducted different MR analytic methods: inverse variance weighted (IVW), weighted median, MR-RAPS and MR-PRESSO and the reliability of the results was increased using sensitivity analyses such as MR-Egger, Cochrane's Q test, MR-RAPS, MR-PRESSO and leave-one-out.	Power calculation was performed and power issue was considered.	**5 (High quality)**

				instruments were greater than 10.	robustness of significant results;<br>2) Study participants were restricted to individuals of European ancestry.			
**Wen Y**	**2025**	Genetic evidence of the causal relationships between psychiatric disorders and cardiovascular diseases.	39752762	**Satisfied (score = 2)**<br><br>1) Genetic instruments were at genome-wide significance with the exposure ($p < 5 \times 10^{-8}$);<br>2) The F-statistics of genetic instruments were greater than 10.	**Satisfied (score = 2)**<br><br>1) weighted mode method was used to detect abnormal SNPs affecting the results and re-calibrating the results after removing the outliers;<br>2) Study participants were restricted to individuals of European ancestry.	**Satisfied (score = 1)**<br><br>1) The study conducted different MR analytic methods: MW-IVW, and for sensitivity analysis, robust adjusted profile score (RAPS), inverse variance weighted (IVW), weighted median, and weighted mode were employed as sensitivity analysis methods to assess the robustness of findings; their validitity was assessed using Cochran's and the MR-Egger intercept test.	Power calculation was not performed and power issue was considered in discussion.	**5 (High quality)**
**Xiang W**	**2025**	Causal association between mental disorders and cerebrovascular diseases: Evidence from Mendelian randomization study.	39271072	**Satisfied (score = 2)**<br><br>1) Genetic instruments were at genome-wide significance with the exposure ($p < 5 \times 10^{-8}$);<br>2) The F-statistics of	**Satisfied (score = 2)**<br><br>1) MR-PRESSO method was used to detect abnormal SNPs affecting the results and re-calibrating the results after removing the outliers; the presence of outlier SNPs	**Satisfied (score = 1)**<br><br>1) The study conducted different MR analytic methods: MR-Egger, weighted median (WM), inverse variance weighted (IVW), simple mode (SM), weighted mode (WME) to assess causality; the intercept term of MR-Egger regression was used to determine whether there was pleiotropy at the gene level, and the	Power calculation was not performed and power issue was not considered.	**5 (High quality)**

				genetic instruments were greater than 10.	was also detected by drawing funnel plots;<br>2) Study participants were restricted to individuals of European ancestry.	Cochran's Q test was used to evaluate the heterogeneity among SNPs; sensitivity analyses were performed to assess the stability and confidence of the results, to explore potential biases such as pleiotropy of genes or heterogeneity of the data, and to assess whether an IV significantly affected the outcome variable.		
**Yu Y**	**2024**	A Mendelian randomization study of the effect of mental disorders on cardiovascular disease.	38887450	**Satisfied (score = 2)**<br><br>1) Genetic instruments were at genome-wide significance with the exposure ($p < 5 \times 10^{-8}$);<br>2) The F-statistics of genetic instruments were greater than 10.	**Satisfied (score = 2)**<br><br>1) SNPs potentially associated with the outcome phenotypes were excluded by the PhenoScanner check; MR-PRESSO method was used to detect abnormal SNPs affecting the results and re-calibrating the results after removing the outliers; 2) Study participants were restricted to individuals of European ancestry.	**Satisfied (score = 1)**<br><br>1) The study conducted different MR analytic methods: inverse variance weighted (IVW), weighted median, MR Egger; pleiotropy was assessed using MR-Egger intercept analysis, heterogeneity was assessed using Cochran's, leave-one-out sensitivity analysis was used to assess the robustness of the results and to clarify the presence of individual SNPs that significantly affected the pooled results.	Power calculation was not performed and power issue was not considered.	**5 (High quality)**

**Zheng Z**	**2025**	Causality Between ADHD, ASD, and CVDs: A Two-Step, Two-Sample Mendelian Randomization Investigation.	39402923	**Satisfied (score = 2)**<br>1) Genetic instruments were at genome-wide significance with the exposure ($p < 5 \times 10^{-8}$);<br>2) The F-statistics of genetic instruments were greater than 10.	**Satisfied (score = 2)**<br>1) MR-PRESSO method was used to detect abnormal SNPs affecting the results and re-calibrating the results after removing the outliers; the causal link between the mediators and the risk of CVDs causally associated with ASD or ADHD were evaluated - multiple exposures in the MR analysis were included, accounting for potential confounders and providing a more accurate estimate of the causal effect;<br>2) Study participants were restricted to individuals of European ancestry.	**Satisfied (score = 1)**<br>1) The study conducted different MR analytic methods: inverse variance weighted (IVW), weighted median, MR Egger and MR-PRESSO; pleiotropy was assessed using MR-Egger intercept analysis, heterogeneity was assessed using Cochran's test.	Power calculation was not performed and power issue was not considered.	**5 (High quality)**

ADHD - attention deficit hyperactivity disorder; AF - atrial fibrillation; AIS - any ischemic stroke; AS - any stroke; ASD - autism spectrum disorder; CAD - coronary artery disease; CES - cardioembolic stroke; CHD - congenital heart disease; CVD - cardiovascular disease; HF - heart failure; IVW - inverse variance weighted; LAS -

large-artery atherosclerotic stroke; MI - myocardial infarction; MR - Mendelian randomization; MW-IVW - variance weighted with modified weights; SNPs - single nucleotide polymorphisms; SVS - small-vessel stroke.
